# Building community capacity to stimulate physical activity and dietary behavior in Dutch secondary schools: Evaluation of the FLASH intervention using the REAIM framework

**DOI:** 10.3389/fpubh.2022.926465

**Published:** 2022-08-03

**Authors:** Bonnie Maria van Dongen, Inge Maria de Vries, Monica Antonia Maria Ridder, Michiel de Boer, Ingrid Hendrika Margaretha Steenhuis, Carry Mira Renders

**Affiliations:** ^1^Department of Health Sciences, Faculty of Science, Amsterdam Public Health Research Institute, Vrije Universiteit Amsterdam, Amsterdam, Netherlands; ^2^Human Movement and Education Division, Windesheim University of Applied Sciences, Zwolle, Netherlands; ^3^Department of General Practice and Elderly Care, University Medical Center Groningen, Groningen, Netherlands; ^4^Department of Healthy Society, Windesheim University of Applied Sciences, Zwolle, Netherlands

**Keywords:** community capacity, health-promoting schools, implementation, mixed methods, adolescents, physical activity, dietary behavior

## Abstract

**Background:**

Building community capacity in secondary schools is a promising strategy for the sustainable implementation of school-based health promotion. The Fit Lifestyle at School and at Home (FLASH) intervention explored how building community capacity works for the prevention of overweight following four strategies: leadership, participatory school culture, tailored health-promotion activities, and local networks. This study evaluates the intervention's impact on community capacity and capacity-building processes over a period of 3 years, as well as its effects on adolescents' BMI and waist circumference.

**Methods:**

A mixed-methods design guided by the RE-AIM framework was used. Impact on community capacity was evaluated with semi-structured interviews at the start and end of the intervention and analyzed using an anchored coding scale. Capacity-building processes were evaluated using interviews, journals, questionnaires, and the minutes of meetings. The effects on BMI z-scores and waist circumference were evaluated using a quasi-experimental design comparing an intervention (IG) and reference group (RG), based on multi-level analyses.

**Results:**

Community capacity improved across all intervention schools but varied between capacity-building strategies. Leadership recorded the greatest improvements, aided by the appointment of Healthy School Coordinators, who increasingly focused on coordinating processes and fostering collaborations. Participatory school culture also improved through the adoption and implementation of participatory methods and a general increase in awareness concerning the importance of the Healthy School approach. Although additional health-promotion activities were implemented, stakeholders struggled with tailoring these to the specific dynamics of their schools. Limited improvements were observed in setting-up local networks that could help schools encourage healthy behavior among pupils. Differences in BMI z-scores between IG and RG over the total sample were negligible whereas waist circumference increased slightly more in IG (0.99 cm, 95% CI [.04; 1.93]). However, differences were inconsistent over time and between cohorts.

**Conclusions:**

This study highlights the potential of building community capacity. It emphasizes that this is a process in which stakeholders must become acquainted with new leadership roles and responsibilities. To navigate this process, schools need support in improving communication, establishing local networks, and sustaining capacity-building efforts in school policy.

**Trial registration:**

ISRCTN67201841; date registered: 09/05/2019, retrospectively registered.

## Introduction

Adolescence is a critical period for stimulating healthy physical activity (PA) and dietary behavior, given the vulnerability of adolescents to unhealthy behaviors that are important determinants of chronic conditions, including obesity, cardio-vascular disease and diabetes (1). Moreover, obesity established during this period often tracks into adulthood (2). Schools are important settings for promoting healthy behavior among adolescents, as they spend large amounts of time in these locations, which serve as their primary settings for learning and development (3, 4). Schools are increasingly applying integral whole-school approaches to health promotion, combining classroom health education, school health policies, a physical and social environment that stimulates healthy choices, and relationships between school, home, and the local community—all with the objective of supporting healthy behavior (5). The sustainable implementation of an integral approach in day-to-day practice remains challenging for schools, as staff members, pupils and parents often feel only a limited sense of ownership over specific activities or interventions (6, 7). In addition, schools have difficulty tailoring evidence-based interventions to their own specific contexts and populations (8).

The application of a community-based approach based on the principles of an integral approach could potentially foster sustainable implementation of interventions and decrease the prevalence of overweight and obesity among adolescents (9, 10). Community members are encouraged to cooperate in the creation of a healthy school community, where health-promotion activities and interventions can be embedded within the complex, dynamic systems of their specific schools (11). To implement such an approach, stakeholders need to build community capacity to ensure continuous improvement (12). Building community capacity entails developing knowledge, skills, ownership, leadership, structures, and systems at the individual and organizational level (13, 14). Such efforts have proven effective in school settings for decreasing the prevalence of overweight and obesity among Australian adolescents (15). However, little is known about whether and how building community capacity in the school setting works as a strategy for overweight prevention in other countries (16).

In the Netherlands, many secondary schools apply the integral Dutch Healthy School approach, a translation of the whole-school approach for the Dutch context advocated by the WHO. Schools can earn theme certificates on health topics by providing health education and having health policies, a healthy physical and social environment, and a referral system in place (17). Schools most often work on health topics concerning physical activity and nutrition (18). Despite the initial success of this approach, issues regarding ownership, participation, and tailoring of interventions remain barriers to sustainable implementation (19).

The Fit Lifestyle at School and at Home (FLASH) intervention was developed to identify ways of building community capacity in Dutch schools in order to design and implement integrated health-promoting activities on healthy dietary and physical activity behaviors of adolescents. This intervention involved four secondary schools, each operating under a different context (20). Processes of co-creation between research and practice were a central component of this intervention, thereby ensuring exchange between evidence-based intervention strategies and the contextual opportunities of each school. Based on the limited research on strategies for capacity-building in school communities, we used the Community Readiness to Change (CRC) method to focus on four specific capacity-building strategies. This method assists communities in the implementation of effective and broadly supported programs, recognizing the need for changes within and between individuals and organizational structures (21). The strategies involved are: (1) identifying and motivating leaders who are able to take charge of the process of creating a Healthy School; (2) promoting a participatory school culture in order to develop broadly supported goals for the Healthy School; (3) designing and implementing tailored health-promotion activities that fit within the Dutch Healthy School approach in order to achieve these goals; and (4) creating a local network of collaborations and resources to ensure continuation.

In this study, we assessed the impact of the FLASH intervention on community capacity and evaluate the capacity-building processes for each intervention school, thereby generating insight into contextual factors that affect the adoption and implementation of this community-based approach. In addition, we investigated the effects of the intervention on the BMI and waist circumference of adolescents.

## Methods

### Study design

We adopted a mixed-methods design to evaluate the FLASH intervention to encompass not only the impact on changes in community capacity and health outcomes in adolescents, but also to gain insight in capacity-building processes. The FLASH intervention took place between September 2016 and July 2019 in four secondary schools in the Netherlands, each with their own dynamic and specific context. The evaluation study was conducted from September 2016 through March 2020. The process of building community capacity for creating a healthy school community, as well as its evaluation, followed an adaptive approach to enable changes in the system to be captured and accommodated, and to allow for feedback and emergent outcomes (22).

This evaluation was guided by the RE-AIM (Reach, Effectiveness, Adoption, Implementation, Maintenance) framework, focusing on design, dissemination and implementation processes (23). Within this framework, there is an growing focus on the value of qualitative data in addition to quantitative data to provide a better understanding of what happened as well as the “how” and “why”, which is also in line with the purpose of the current study (24).

Impact on community capacity was evaluated with semi-structured interviews based on the CRC method (21). Interviews were held at the start and end of the intervention, and they were analyzed using an anchored coding scale in order to create a score for community capacity. Capacity-building processes were evaluated using interviews, journals, questionnaires, and minutes of meetings held throughout the intervention period. The intervention's effect on the BMI and waist circumference of adolescents was evaluated using a quasi-experimental design comparing the intervention group to a reference group. The main results of the intervention are described in this paper. Elsewhere, we will elaborate on the capacity-building processes and on lessons learned on how to encourage these processes in secondary schools. Ethical approval was provided by the Dutch Medical Research Involving Human Subjects Act (Medical Ethics Committee of Amsterdam UMC, reference number 2016.352).

### Setting and population

The FLASH intervention builds on the integral Dutch Healthy School approach (25). Stakeholders in the school community are pupils, staff, and parents. The intervention was aimed specifically at the lower educational tracks, as a relatively large share of pupils who are at risk for unhealthy PA and dietary behavior are enrolled in these (26). In the Dutch context, these tracks (also known as “streams”) are jointly referred to as pre-vocational secondary education (*vmbo*)^1^ (27). Four schools belonging to the same regional educational partnership in the northeastern region of the Netherlands were recruited. Schools were eligible to participate if they were willing to commit to the intervention and evaluation for 4 years. In addition, the school board needed to be willing to facilitate new health-promotion activities and to appoint a staff member to coordinate the FLASH intervention. For the quasi-experimental study, four reference schools were recruited based on characteristics matching those of the intervention schools (e.g., surroundings, size, educational streams, and familiarity with the Healthy School approach). To prevent contamination of the results during the intervention, schools belonging to the same educational partnership as the intervention schools were excluded.

### FLASH intervention

**Figure 1:**
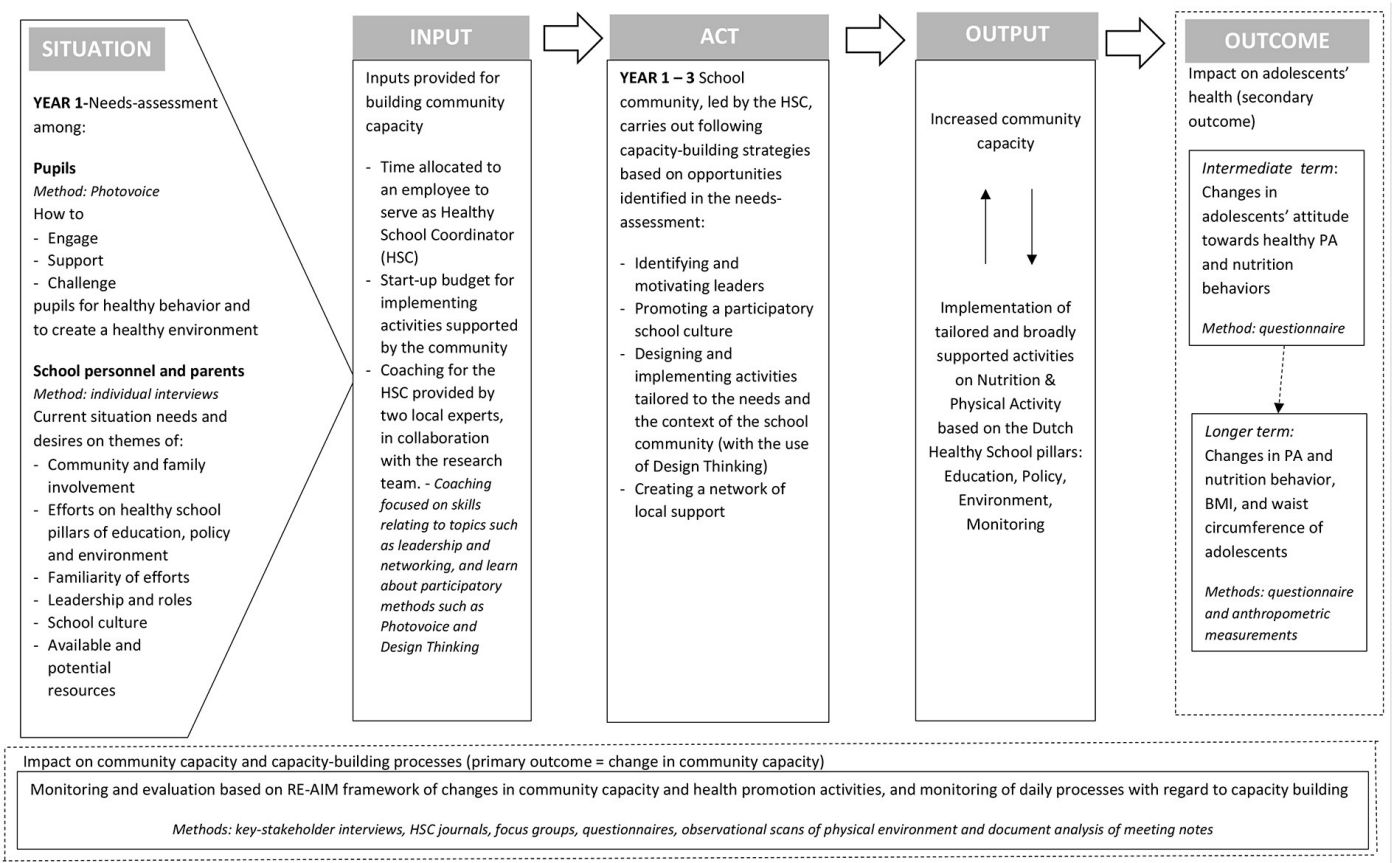
Logical model of the FLASH intervention.

The focus of the FLASH intervention was to implement the four capacity-building strategies into the daily practice of four intervention schools. The intervention was designed based on the logical model in Figure 1 (20). Building community capacity was considered a continuous and context-specific process for each intervention school, because schools in the Netherlands have a lot of autonomy and can differ for example in educational vision, culture, number of students (28). Based on a needs-assessment among pupils, school personnel and parents to map their context-specific situation, stakeholders in each school community were supposed to act on identifying and motivating leaders, promoting a participatory school-culture, designing and implementing tailored activities and creating an active network. By working on these strategies, we expected community capacity of schools to increase (output) (29). Increased community capacity potentially leads to the creation of (improved) health-promotion activities that are tailored to the needs, opportunities and context of a school, follow the principals of the whole-school approach, and can be sustained over time. Building community capacity and implementing tailored health-promotion activities were treated as two reciprocal processes that can influence each other: successful activities can increase community capacity, and increased community capacity can help the school community to replace or adjust less successful activities. Moreover we hypothesized that this will eventually lead to changes in behavior of pupils in the intermediate term and in anthropometric measures in the longer term (outcome).

To empower intervention schools in capacity-building processes, several inputs were provided: time allocated to an employee who served as a Healthy School Coordinator (HSC), a start-up budget for implementing activities supported by the school community, and coaching for the HSC provided by two local experts in the field of health promotion and education. These experts had knowledge of evidence-based interventions involving the Healthy School approach, and they had local connections to organizations related to health promotion. The experts and research team collaborated closely in the coaching of HSCs, thereby establishing an iterative process of co-creation. Coaching consisted of both individual and group sessions where HSCs could exchange experiences, learn skills relating to various topics (e.g., leadership and networking), and learn about methods for participation (Photovoice and Design Thinking). Both experts had experience with supporting schools in the Dutch Healthy School approach as this was part of their regular job-description. Their additional and new task in the FLASH intervention was to guide the HSC specifically in the process of creating a healthy school community.

Schools engaged in capacity-building processes throughout three phases. In the first phase, stakeholders in the school community started to establish and appoint leadership roles, had conversations about the responsibilities of leaders, and set up networks that could support leaders. These efforts continued throughout the entire intervention (Years 1–3). At the same time, the needs and wants of pupils, staff, and parents were mapped using interviews and Photovoice, including the identification of opportunities for health-promotion activities (Year 1). In the second phase, the HSCs were stimulated to organize Design Thinking sessions with community members in order to create context-specific solutions and action plans that make use of evidence-based techniques and fit within the Dutch Healthy School approach (Year 2). In the third phase, leaders were responsible for carrying out and evaluating action plans and activities (Year 3). The schools were also encouraged to discuss how working activities could be sustained or adjusted [for a detailed description of FLASH see (20)].

Reference schools were allowed to participate in the regular Dutch Healthy School approach. All Dutch schools are encouraged to voluntarily adopt this approach and make use of the support of a regional health promotion expert. Central in this approach is the execution of health-promotion activities based on four pillars (education, environment, policy, and signaling) on a chosen health topic such as nutrition or PA. The difference with intervention schools mainly lies in the process of building community capacity in order to create a broadly supported and tailored healthy school community.

### Mixed methods

Evaluation outcomes were based on the RE-AIM framework (23). An overview of the outcomes, methods, and analysis for each element is provided in Table 1. All participants signed an informed consent form when they entered the study. Pupils were only allowed to participate if their parents/guardians provided written consent as well.

**Table 1:** Overview of outcomes, methods, and analysis for each RE-AIM element.

**RE-AIM Element**	**Outcomes**	**Methods**	**Analysis**
**REACH**—*potential reach based on number of pupils throughout the intervention*	Overview of characteristics of intervention schools: school size, type of population, school environment	*HSC journals and interviews:* Questions about starting position with regard to the Dutch Healthy School approach and school characteristics	Descriptive analysis and thematic coding
**EFFECTIVENESS**—*impact on community capacity and pupils' health*	Changes in: - Overall community capacity score for each school - Each of the six dimensions of community capacity	*Community Capacity interviews:* Semi-structured (topics on the 6 dimensions of the CRC method), with 6-8 key stakeholders per school at the start and end of the intervention	Anchored coding scale
	Changes in: - BMI, BMI z-scores and waist circumference - Health behavior	*Quasi-experimental study:* 4 intervention schools and 4 control schools; measurement rounds in 2016, 2017, 2018, and 2019	Longitudinal multilevel analysis and descriptive analysis
**ADOPTION**—*willingness to work on capacity-building strategies based on intervention inputs*	*Strategy 1: Leadership* - Willingness to facilitate an individual as HSC	*Minutes of meetings:* Overview of the availability of the HSC throughout the intervention	Thematic coding
	*Strategy 2: Participatory school culture* - Willingness to use participatory methods	*Minutes of meetings:* Records with regard to willingness of schools to organize a Design Thinking session and Photovoice lessons	Thematic coding
	*Strategy 3: Tailored activities* - Willingness to create action plans based on ideas from Design Thinking session - Willingness to initiate new tailored activities	*Minutes of meetings:* Records on whether schools were willing to create action plans based on ideas from Design Thinking sessions. Records on whether schools were willing to create new activities or adjusted existing activities that fit within the Dutch Healthy School approach	Thematic coding
	*Strategy 4: Local networks*- Willingness of local experts - Willingness to organize coaching sessions	*Minutes of meetings:* Overview of the availability of the local expert and the number of coaching sessions initiated by these experts	Thematic coding
**IMPLEMENTATION**—*extent to which intended actions about each capacity-building strategy were implemented*	*Strategy 1: Leadership* - Extent to which the hours available to HSCs were used - Extent to which HSCs were able to motivate community members for leadership roles -Extent to which HSCs used coaching from local experts (individual and group coaching sessions)	*Community Capacity interviews:* Questions about experiences relating to motivating stakeholders to take up leadership in general, as well as in specific activities *HSC journals and interviews:* Questions about their experiences in this role throughout the intervention	Thematic coding
	*Strategy 2: Participatory school culture* - Extent to which community members participated in the creation of tailored ideas during Design Thinking sessions - Extent to which pupils participated in the creation of tailored ideas during Photovoice sessions	*Minutes of meetings:* Records about the extent to which Photovoice lessons and Design Thinking sessions were implemented, including attendance and ideas arising from these methods	Thematic coding
	*Strategy 3: tailored activities* - Extent to which action plans were carried out as intended in each school - Extent to which Healthy School activities were initiated or adapted	*Community Capacity and HSC interviews:* Questions about the use of the implementation budget and how this activity came to be, as well as questions about other Healthy School activities relating to each pillar that took place during the intervention *Minutes of meetings:* records about the process of developing action plans and rewarding implementation budget	Thematic coding
	*Strategy 4: Local networks* - Extent to which schools initiated collaborations with local partners	*Community Capacity interviews:* Questions about whether collaborations with local organizations were initiated and the relative success of these collaborations *HSC journals and interviews:* Questions in journals about HSCs' attendance at and rating of coaching sessions, as well as interview questions about how their perceptions of individual coaching	Thematic coding
**MAINTENANCE**—*extent to which stakeholders intend to continue working with a community-based approach*	Extent to which management and HSCs are planning to continue new activities and/or inputs	*Community capacity interviews:* Questions added to the interviews with HSCs and managers at the end of the intervention about potential continuation	Thematic coding
	Extent to which local health (and/or other) organizations are willing to continue supporting schools	*Maintenance interviews with local experts:* Semi-structured interviews with stakeholders in organizations, who are responsible for continued support and who were involved in the FLASH intervention; conducted in the third year	Thematic coding

#### Community capacity interviews

In each intervention school, six to eight semi-structured interviews were held at the start and end of the FLASH intervention based on the CRC-method (21). Following this method, we included stakeholders with varying roles who had knowledge about the school community. By including a cross-section of individuals in the school community in in-depth interviews, a multi-faceted and detailed picture of the schools' situation was obtained (30). At both time-points, the following stakeholders were recruited: HSC (T1:*N* = 4, T2:*N* = 4), school directors or managers (T1:*N* = 4, T2:*N* = 4), teachers (T1:*N* = 8, T2:*N* = 9), support staff (e.g., canteen employees or janitors) (T1:*N* = 4, T2:*N* = 4), and parents (T1:*N* = 3, T2:*N* = 3). Purposive sampling was applied with the assistance of the HSCs, who were acquainted with key stakeholders in their communities. All interviews were audio-recorded and transcribed verbatim.

The CRC method was used to operationalize the four capacity-building strategies that are central to the FLASH intervention (Table 2). This method differentiates six dimensions of community readiness: activities, visibility of activities, knowledge about local prevalence to prioritize activities, leadership, community culture/climate, and resources/local collaborations (21). Strategies 1 (leadership), 2 (participatory school culture), and 4 (local networks) correspond directly to specific CRC dimensions. Strategy 3 (tailored activities) combines the three CRC dimensions—activity, visibility, and local prevalence—as they all relate to the design and implementation of context-specific health-promotion activities, as well as to the existing Dutch Healthy School approach to the development of structural activities.

**Table 2:** CRC method as indicators for community capacity strategies.

**Strategy**	**Indicator(s) based on CRC method**	**Example questions**
S1: Leadership	Leadership dimension: Readiness of leaders and other influential stakeholders in the school community to commit to and facilitate actions on this theme^*a*^	- Who are leaders in your community, and are they willing to support action/take action? - How are responsibilities distributed between leaders and collaborations on this theme organized and facilitated?
S2: Participatory school culture	Culture/climate dimension: Attitude of community members toward the importance of this theme and their readiness to facilitate participation when designing solutions that suit the needs of their communities	- According to community members, how important is it to take action on this theme? - Are community members involved in the design of actions and activities? Are they willing to participate in designing these actions and activities?
S3: Tailored activities	Activities dimension: Extent to which structural activities take place (fitted to the Healthy School approach) for which the community has a sense of ownership regarding this theme	- Which activities take place in this school (i.e., education, the physical environment, and policy pillars)? - How long have these activities existed and to what extent do they suit this school?
	Familiarity dimension: Extent to which community members are familiar with actions and activities taking place regarding this theme	- To what extent are community members familiar with the Healthy School approach and activities on this theme (social environment pillar)? - What actions does this school take to enhance familiarity?
	Knowledge about the prevalence dimension: Extent to which actions and activities are based on local prevalence and knowledge about this theme	- Is a system in place to monitor the health, behavior, and knowledge of pupils (signaling pillar) in this specific school on this theme? - To what extent is this information used to tailor actions and activities on this theme?
S4: Local networks	Resources and local collaborations dimension: Extent to which local collaborations and resources are in place to sustain the actions and activities on this theme	- Are resources or collaborations with local organizations in place that can ensure continued support for activities, either by providing information, guidance, or means (e.g., funding, materials)?

a Theme refers to promoting healthy physical activity and dietary behavior among vmbo-pupils.

#### HSC journals and interviews

Each HSC filled out a digital journal approximately once every 2 months during the first two years of the intervention. Questions related to attendance at coaching sessions, time investment of allocated hours, and experiences with the role of HSC. At the start of the intervention, HSCs answered questions about their schools' current Dutch Healthy School efforts. The status of the food environment was recorded using a canteen scan (31). Each HSC participated in an interview at the start and end of the intervention concerning the school context, characteristics, expectations, experiences, and reflections throughout the intervention. All interviews were audio-recorded and transcribed verbatim.

#### Minutes of meetings

Minutes were kept of the following meetings: project team (the research team and two local experts), coaching sessions, and contact moments between researchers and HSCs or experts. These data sources provided in-depth qualitative information about experiences throughout the FLASH intervention.

#### Maintenance interviews with local experts

In the third year of the intervention semi-structured interviews were held with the two experts concerning their coaching experiences, as well as with managers in the organizations of these experts (*N* = 5) about embedding components of the intervention and the coaching role into their organizations. The interview guide was based on the Measurement Instrument for Determinants of Innovations framework, a systematically designed tool to measure determinants of innovations that may affect their implementation (32). The interviews were audio-recorded and transcribed verbatim.

#### Quasi-experimental study

A quasi-experimental study was conducted among pupils enrolled in secondary vocational education in four intervention and four reference schools. Participants were recruited among second-year pupils in September 2016 (Cohort A), September 2017 (Cohort B), and September 2018 (Cohort C). Informed written consent was obtained from both parents and pupils. Four measurement rounds were conducted, with participants being followed over time until they graduated (fourth-year on average) or until the end of the study. Anthropometric measurements (secondary outcomes) including weight (nearest 0.1 kg), height, and waist circumference (both nearest 0.1 cm) were assessed among participants, as measured by trained research assistants according to a protocol. Weight and height were used to calculate BMI (kg/m^2^) and transformed into z-scores based on Dutch reference values (33). In addition, a self-reported questionnaire, partly based on a validated tool (34), was administered digitally during school hours to obtain information on demographics and health behavior. Outcomes included were: PA behavior based on compliance to the Dutch norm of healthy PA, screen time, dietary behavior based on consumption of water, sugar sweetened beverages, breakfast, fruit, vegetable and snacks, and attitude toward these behaviors (see Appendix I for these secondary outcomes). For a detailed description see (20).

### Analyses

#### Anchored coding to assess community capacity

The anchored coding system of the CRC method was used to create a community capacity score for each intervention school. This system has previously been used and validated as an indicator of community capacity (15, 21). Coding was performed independently by two researchers and results were discussed. A third researcher was consulted if no consensus was reached. Reflective diaries were kept in order to evaluate subjective views.

**Figure 2:**
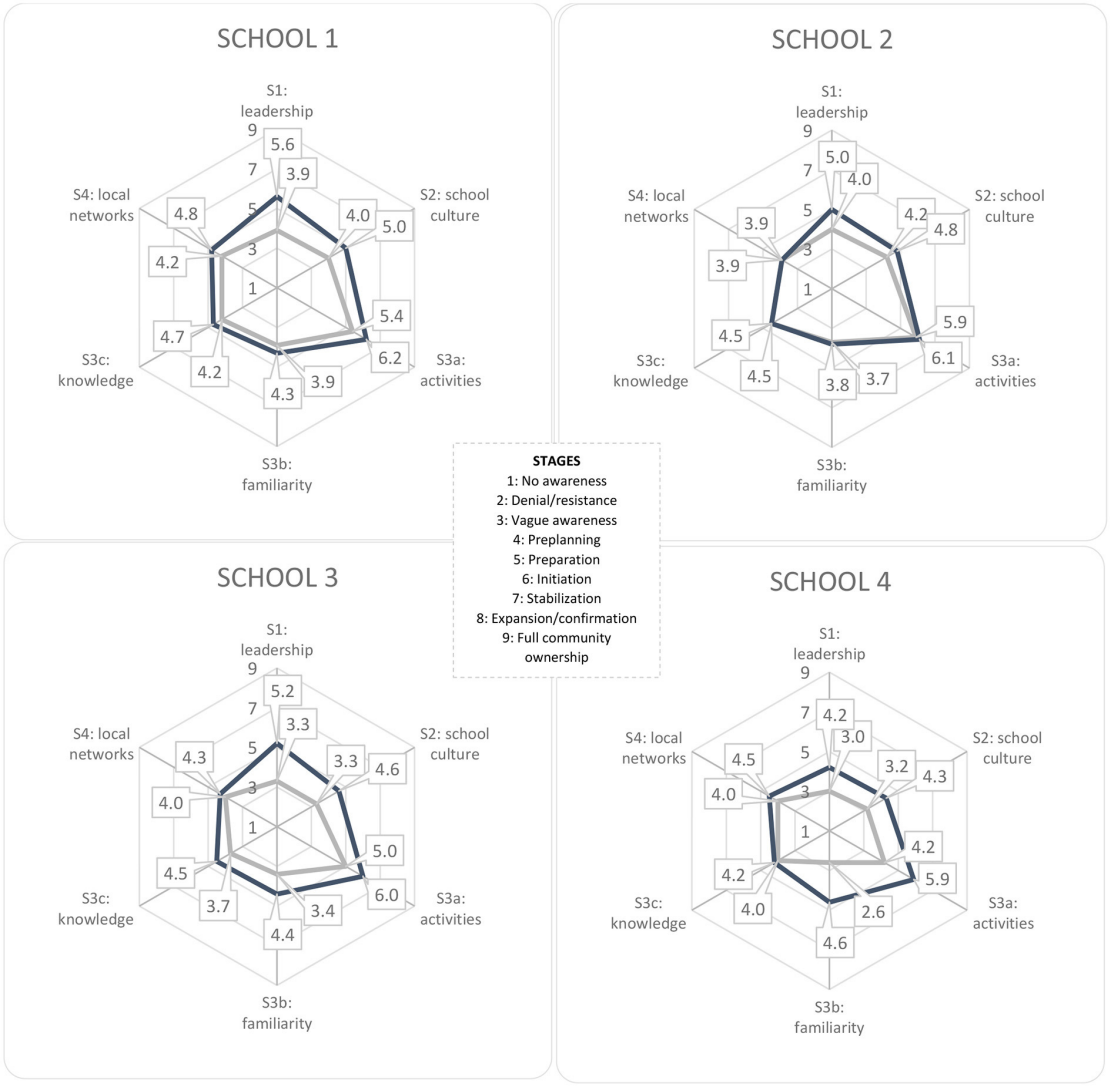
Changes in community capacity per intervention school.

Each interview was coded on segments that reflected the current status of the community for each indicator (Table 2). Segments were scored on a stage of readiness, ranging from 1 (no awareness) to 9 (high level of community ownership) (Figure 2). For each interview, coded segments were converted into an average score for each indicator that reflected the community's stage of readiness. If consensus between researchers was reached, the individual scores for each indicator were summed and divided by the number of respondents at each school. For each school, the average scores for each indicator were summed and divided by six at both measurement points to create an overall score for community capacity. This yielded a score for change in community capacity for each indicator and overall for each school.

#### Thematic coding to assess impact on daily capacity-building processes

Thematic analyses, based on the elements of the RE-AIM framework, were conducted on qualitative data derived from interviews and minutes of meetings using MAXQDA 2018. Coding was done independently by two researchers. Coding and results were discussed by the researchers and the research team.

#### Quantitative analysis to assess changes in adolescents' health

The impact of the FLASH intervention on BMI z-scores and waist circumference was analyzed using multilevel linear regression analyses in STATA-16. Analyses were performed on the total sample and on each cohort separately, with data collected at baseline, follow-up (T1) and a secondary follow-up (T2). Longitudinal multilevel analyses were performed on the total sample and on Cohorts A and B separately, adjusted for baseline differences. To assess the effects of the intervention over time, an interaction term for time was added to each model as a categorical variable. To correct for cluster effects at the school level and for repeated measurements within participants, a three-level hierarchical data structure was applied. The goodness of fit of the models were compared using the likelihood-ratio test. For the analyses on Cohort C, a multilevel analysis was performed, based on a two-level hierarchical data structure (pupils within schools), adjusted for baseline differences.

Adjusted models included sex (male/female), educational level (“learning pathway”/“profile;” see Note 1), and migration background (no migration or Western migration background vs. non-Western migration background). Non-Western migration background was defined as at least one parent had a migration background from countries in Africa, Latin-America, Asia, or Turkey (35). Results are described as mean differences over time with 95% confidence intervals. For outcomes concerning physical activity and dietary behavior, descriptive statistics were calculated using IBM SPSS Statistics 26.

## Results

At the start of the FLASH intervention, all intervention schools were acquainted and working with the integral Dutch Healthy School approach to varying degrees, with Schools 1–3 being further along than School 4. Detailed information including contextual factors operating in each school throughout the intervention is provided in Appendix II. All schools reported having more difficulty with activities focusing on healthy dietary behavior than with those focusing on PA. An overview of the FLASH intervention's impact on each school is provided in Table 3, broken down by RE-AIM element.

**Table 3:** Overview of results based on RE-AIM elements.

	**School 1**	**School 2**	**School 3**	**School 4**
**Reach—school population:**				
*School environment*	Comprehensive school, located in rural area (see Appendix II for contextual information)	Comprehensive school, located in city center (see Appendix II for contextual information)	Exclusively offers “profiles” sub-stream of *vmbo* track, located in city center (see Appendix II for contextual information)	Exclusively offers “profiles” sub-stream of *vmbo* track, located in rural area (see Appendix II for contextual information)
*Size of school in N of pupils: total N (N pre-vocational)*	305 (219) in September 2016 297 (213) in September 2017 294 (204) in September 2018	711 (221) in September 2016 842 (245) in September 2017 962 (286) in September 2018	520 in September 2016 638 in September 2017 612 in September 2018	228 in September 2016 199 in September 2017 160 in September 2018
**Effectiveness—change in:**				
*Community capacity*	Overall capacity change: 4.3 → 5.1 Per dimension: See Figure 2	Overall capacity change: 4.4 → 4.7 Per dimension: See Figure 2	Overall capacity change: 3.8 → 4.8 Per dimension: See Figure 2	Overall capacity change: 3.5 → 4.6 Per dimension: See Figure 2
*Pupils' health & behavior*	See Table 4 and Appendix I			
**Adoption—willingness to/from**:				
***Strategy 1: Identifying leadership***				
*Facilitate HSC*	One HSC was facilitated continuously for the duration of intervention.	One HSC was facilitated continuously for the duration of intervention.	One HSC was facilitated continuously for the first two years of the intervention. A change in HSC took place in Year 3. The new HSC was engaged in the intervention for $\frac{3}{4}$ of Year 3.	One HSC was facilitated continuously in Year 1. A change of HSC took place in Year 2. The new HSC was facilitated continuously in Years 2 and 3.
***Strategy 2: Creating a participatory school culture***				
*Use participatory methods*	Design thinking: Yes, start of Year 3 Photovoice: Yes, in Years 1 and 2	Design thinking: Yes, end of Year 2 Photovoice: Yes, in Years 1 and 2	Design thinking: Yes, start of Year 3 Photovoice: Yes, in Years 1 and 2	Design thinking: Yes, end of Year 2 Photovoice: Yes, in Years 1 and 2
***Strategy 3: Designing tailored activities***				
*Develop action plan for implementation budget*	Yes, together with colleague, and based on input from DT session	Yes, together with colleague, and based on input from DT session	Yes, but the first action plan was rejected based on limited input from DT session and lack of integral approach. Adjustments were made.	Yes, together with colleague, and based on input from DT session
*Facilitate changes for the Healthy School approach*	Yes, but resources limited due to small size of the school	Yes, but priorities shifted due to increasing pupil numbers	Yes, by a manager and teacher. Concerns were expressed with regard to willingness of canteen staff.	Yes, but with the note that certain changes require approval of all three schools in the building
***Strategy 4: Creating local networks***				
*From local organizations*	Expert from educational organization: Role was facilitated throughout Years 1 and 2 and for 1/3 of Year 3. One person held this position continuously. Expert from municipal health service: One person was facilitated from October through March of Year 1. A new person was facilitated from May in Year 1 until January in Year 3. In all, the experts organized 15 coaching sessions for the HSCs, in collaboration with the principal researchers.			
**Implementation—extent to which:**				
***Strategy 1: Identifying leadership***				
*HSC hours were used*	HSC indicated using allocated weekly hours most of the time	HSC indicated using allocated weekly hours half of the time	HSC indicated often not using allocated weekly hours, due to other priorities	HSC indicated using allocated weekly hours half of the time
*Other leaders were motivated*	See Appendix III	See Appendix III	See Appendix III	See Appendix III
*Coaching sessions were attended*	13 out of 15 (average experience score of 8.3)	15 out of 15 (average experience score of 6.9)	12 out of 15 (average experience score of 7.6)	13 out of 15 (average experience score of 7.5)
***Strategy 2: Creating a participatory school culture***				
*Community participated in Design Thinking sessions*	17 participants in total: 3 teachers, 1 school manager, 2 parents, 7 pupils, the HSC, and 1 local expert	13 participants in total: 3 teachers, 1 team leader, 2 parents, 2 pupils, the HSC, and 1 local expert	22 participants in total: 3 teachers, 1 team leader, 1 PR employee, 2 parents, 7 pupils, the HSC, and 1 local expert	5 participants in total: 1 team leader, 2 parents, 1 pupil, the HSC, and 1 local expert
*pupils participated in Photovoice*	2 second-year classes engaged in 4 2-hour sessions	1 second-year class engaged in 4 2-hour sessions	7 pupils engaged in 3 1-hour sessions	2 second-year classes engaged in 1 afternoon session
***Strategy 3: Designing tailored activities***				
*Action plan was carried out*	Plan was not carried out during the intervention, but an adjusted activity was conducted.	Plan was carried out, but the impact remained limited due to implementation issues concerning reach.	Part of the plan was carried out, aimed largely at incidental activities instead of structural changes.	Plan was largely carried out, with revisions necessitated by contextual factors.
*Additional Healthy School activities were set up*	- A second activity created in the DT session was implemented with school resources. - Changes were made to the school canteen and screen-time policies aimed at reducing sedentary behavior were adopted.	- Changes were made to the nutrition policy. - Successful existing activities were adjusted and continued. - Willingness to facilitate activities in the new pre-vocational school location was limited.	- A water tap was installed. - Changes were attempted in the school canteen and in-house retail shop run by pupils - The school yard was re-designed with school resources.	- A water tap was installed. - Health education and a Healthy School canteen were implemented out in the new location.
***Strategy 4: Creating local networks***				
*Connections were established with local organizations*	- Structural contact with sports organizations by the PE teacher - Structural collaboration with local municipal youth team	- Structural contact with sports organizations by PE teacher, who also uses available sports equipment from the Landstede Group as a resource - Conversation initiated with municipality about physical environment, but with limited impact	- Collaboration with organizations for internships, but no connections established with regard to health promotion - School management in contact with local supermarket about waste reduction, but not about health promotion	- Collaboration initiated between the school and neighborhood sports coaches, but additional support needed to make the collaboration more profitable for both parties
**Maintenance—willingness to maintain:**				
*HSC role*	Yes, but the tasks are embedded within the existing hours of the care coordinator and biology and PE teachers. No additional funds are available.	Yes. The school leader is continuing to facilitate the HSC for 75%. To build on the lessons learned from the intervention, the HSC will collaborate with a PE colleague.	Yes, depending on financial support received from the Dutch Healthy School approach. A Healthy School working group consisting of teachers remains part of the organizational structure.	Undecided. The current HSC remains involved with the Healthy School approach based on financial support received. There are no immediate plans to appoint a separate HSC specifically for the intervention location.
*Activities*	Yes. There is the intention to incorporate the greenhouse project into the curriculum and expand the project by establishing connections with the school canteen. Budget will still be used for standing desks.	Yes. There is the intention to address implementation issues with regard to reach and communication, as well as to have another trial period.	Partly. School leaders made budget available to provide new pupils with water bottles. The biology teacher showed motivation to continue with curriculum adjustments.	Yes. There is the intention to make the staircase challenge an annual event. The biology teacher and the care and welfare teacher are discussing the possibility of extending this with the new curriculum.
*Support from local organizations*	Educational organization: Limited, due to organizational changes Municipal health service: Willing, although training and additional time are needed Other organizations for advisory board: Recognition of the benefits of a community-based approach, but clarity is needed with regard to roles and responsibilities			

### Reach

- Throughout the intervention and evaluation, Reach proved difficult to define in absolute numbers, because this intervention was aimed at instigating desired but unpredictable changes in the school community. Due to the dynamic and complex nature of such systems, adjustments were made to the operationalization in the original design of this study, redefining Reach as “potential reach based on number of pupils”.

At the start of the intervention, Schools 1, 2, and 4 had approximately 200 *vmbo*-pupils each, and School 3 had approximately 500. School 1 and 2 were comprehensive schools with a larger potential reach of 305 pupils (School 1) and 711 pupils (School 2) in total. Stakeholders mentioned that changes to the environment or policy changes will be aimed at the total pupil population. Pupil numbers varied throughout the intervention period, due to pupils leaving school and the influx of new pupils. The number of pupils in School 1 remained stable, while Schools 2 and 3 experienced an increase (+35% total/+29% prevocational and +18% respectively) and School 4 experienced a decline (−30%).

### Effectiveness

- Effectiveness was defined as impact on community capacity and pupils' health.

#### Changes in community capacity

The overall capacity score at the start of the FLASH intervention varied between Stage 3 (vague awareness) and Stage 4 (pre-planning) (see Figure 2). After the intervention, the overall scores had increased to approximately Stage 5 (preparation) in all schools.

For Strategy 1 (leadership) and Strategy 2 (participatory school culture), Schools 1 and 2 scored at approximately Stage 4 (pre-planning), with Schools 3 and 4, respectively, scoring between Stage 3 (vague awareness) and Stage 4 (pre-planning) at the start of the intervention. For Strategy 1 (leadership), the end scores for Schools 1–3 had increased to between Stage 5 (preparation) and Stage 6 (initiation), with those for School 4 increasing to Stage 4 (pre-planning). For Strategy 2 (participatory school culture), the scores for Schools 1–3 had increased to approximately Stage 5 (preparation), with School 4 increasing to Stage 4 (pre-planning).

For Strategy 3A (activities), the initial scores for Schools 1–3 were between Stage 5 (preparation) and Stage 6 (initiation), with School 4 scoring at Stage 4 (pre-planning), reflecting that school's starting position with the Healthy School approach. After the intervention, all schools scored at Stage 6 (initiation). For Strategy 3B (visibility of activities), Schools 1 and 2 scored around Stage 4 (pre-planning), and Schools 3 and 4 scored around Stage 3 (vague awareness). After the FLASH intervention, the scores for Schools 3 and 4 had increased to between Stage 4 (pre-planning) and Stage 5 (preparation), while the scores for Schools 1 and 2 remained approximately the same. For Strategy 3C (knowledge about local prevalence to prioritize activities), the initial scores for all schools were around Stage 4 (pre-planning), and the ending scores for all schools increasing to between Stage 4 (pre-planning) and Stage 5 (preparation).

For Strategy 4 (local networks), the initial scores for all schools were around Stage 4 (pre-planning). The ending scores for Schools 1, 3, and 4 reflected a slight increase to between Stage 4 (pre-planning) and Stage 5 (preparation).

#### Changes in the BMI, waist circumference and health behavior of pupils

Estimated differences in BMI z-scores were small for the total sample and remained stable over time (B-T1: −0.08, 95% CI [−0.19, 0.03], B-T2:−0.09, 95% CI [−0.21, 0.3]) (see Table 4). The estimated difference for waist circumference overall was.99 cm (95% CI [0.04; 1.93]) higher for pupils in intervention schools than for pupils in reference schools, but varied over time (B-T1: 1.40, 95% CI [0.43; 2.37], B-T2:0.13, 95% CI [−1.01; 1.27]). Estimated differences in BMI z-scores and waist-circumference for cohorts separately showed variations between the cohorts for both overall effect and effects over time periods. For example, the estimated difference for the overall effect in BMI z-scores for cohort A was −0.26 (95% CI [−0.41; −0.11]), for cohort B.08 (95% CI [−0.12; 0.25]) and for cohort C −0.02 (95% CI [−0.17; 0.13]).

**Table 4:** Estimated differences in BMI z-scores and waist circumference between pupils of intervention and reference schools.

	**Crude**			**Adjusted** ^***a***^		
	**β**	**95% CI**	***p*-Value**	**β**	**95% CI**	***p*-Value**
**Total sample**
**BMI z-scores (*****N** **=*** **460)**
Baseline—T1	−0.08	[−0.19; 0.03]	0.159	−0.08	[−0.19; 0.03]	0.142
Baseline—T2	−0.09	[−0.21; 0.04]	0.172	−0.09	[−0.21; 0.03]	0.155
Overall effect	−0.09	[−0.21; 0.02]	0.112	−0.09	[−0.19; 0.02]	0.096
**Waist circumference (*****N** **=*** **441)**
Baseline—T1	1.36	[0.41; 2.31]	0.005	1.40	[0.43; 2.37]	0.005
Baseline—T2	0.07	[−1.05; 1.20]	0.898	0.13	[−1.01; 1.27]	0.821
Overall effect	0.93	[0.05; 1.82]	0.039	0.99	[0.04; 1.93]	0.040
**Cohort A** ^***b***^
**BMI** ***z*****-scores (*****N** **=*** **152)**
Baseline—T1	−0.22	[−0.38; −0.06]	0.006	−0.23	[−0.39; −0.08]	0.003
Baseline—T2	−0.27	[−0.44; −0.10]	0.002	−0.28	[−0.45; −0.11]	0.001
Overall effect	−0.25	[−0.40; −0.10]	0.001	−0.26	[−0.41; −0.11]	0.001
**Waist circumference (*****N** **=*** **149)**
Baseline—T1	2.18	[0.29; 4.07]	0.024	2.16	[0.34; 3.97]	0.020
Baseline—T2	1.84	[−0.07; 3.75]	0.060	1.79	[−0.05; 3.64]	0.057
Overall effect	1.95	[0.09; 3.80]	0.040	1.91	[0.16; 3.66]	0.032
**Cohort B** ^***c***^
**BMI** ***z*****-scores (*****N** **=*** **166)**
Baseline—T1	0.05	[−0.16; 0.28]	0.591	0.06	[−0.16; 0.28]	0.591
Baseline—T2	0.08	[−0.13; 0.30]	0.453	0.08	[−0.13; 0.30]	0.454
Overall effect	0.08	[−0.12; 0.27]	0.455	0.08	[−0.12; 0.28]	0.455
**Waist circumference (*****N** **=*** **145)**
Baseline—T1	1.65	[−0.15; 3.47]	0.073	1.70	[−0.01; 3.41]	0.051
Baseline—T2	−1.11	[-2.90; 0.67]	0.222	−1.08	[-2.76; 0.61]	0.211
Overall effect	0.37	[−1.18; 1.93]	0.639	0.44	[−1.06; 1.95]	0.563
**Cohort C** ^***d***^
**BMI** ***z*****-scores (*****N** **=*** **142)**
Baseline—T1	−0.04	[−0.20; 0.12]	0.623	−0.02	[−0.17; 0.13]	0.775
**Waist circumference (*****N** **=*** **147)**
Baseline—T1	−1.04	[-4.11; 2.03]	0.506	−1.14	[-3.63; 1.36]	0.372

a Adjusted for sex, educational level and migration background.

b Measurement rounds were conducted as follows: Baseline measurement in 2016, T1 in 2017, T2 in 2018.

c Measurement rounds were conducted as follows: Baseline measurement in 2017, T1 in 2018, T2 in 2019.

d Measurement rounds were conducted as follows: Baseline measurement in 2018, T1 in 2019.

Descriptive analyses of behavioral outcomes indicated that water consumption in the intervention group improved from 2.3 (1.8) to 3.0 (2.1) glasses a day, while consumption in the reference group remained stable although consumption started at a higher level [T1: 2.7(1.8), T4: 2.8(1.8)]. In both groups, adherence to the Dutch standard for physical activity improved, with the intervention group increasing from 4.8 (1.9) days a week to 5.7 (1.9), and the reference group increasing from 5.1 (1.9) to 5.7 (1.9) days a week. Other outcomes (e.g., fruit and vegetable consumption; snacking behavior; attitude) were similar over time for both groups, as presented in Appendix I.

### Adoption and implementation

- Adoption and implementation processes were running simultaneously during this intervention. In the original design of this study, adoption was seen as a one-moment decision. However, throughout the intervention adoption turned out to be an ongoing process and was supported by the guidance schools received. Therefore, we redefined adoption from the original design as “the willingness to work on capacity-building strategies based on intervention inputs”. The implementation process was not an independent activity of the school community, but was supported by the local experts, principal researcher and the inputs of the intervention (e.g., start-up budget). Hence, implementation was redefined as “the extent to which intended actions about each capacity-building strategy were implemented”. Adoption and Implementation were also more specifically defined for each strategy.

#### Strategy 1: Leadership

- Adoption: facilitation of an individual as HSC.
- Implementation: available hours to HSCs, ability to motivate community members for leadership roles, individual and group coaching for HSCs.

Three schools had an HSC throughout the FLASH intervention. In School 3, no HSC was available for the last 3 months. In Schools 3 and 4, the HSC role was transferred to a different person, due to shifting teaching responsibilities. The HSCs reported that the intervention had influenced the way in which they implemented their leadership role. Instead of simply organizing health-promotion activities, their responsibilities shifted toward bringing people together, as they perceived this to be helpful in creating ownership. They remained hesitant to ask colleagues, parents, and pupils to take on leadership roles, due to the anticipated additional work pressure and limited resources to ease this pressure. For this reason, they were more inclined to seek opportunities within existing leadership structures. The HSCs tried to involve parents and pupils through the existing councils, and they relied on responsible colleagues for entry into these bodies. To inspire other colleagues for leadership roles, the HSCs first turned to those who were already fulfilling health-related tasks (e.g., PE or biology teachers). They also initiated connections with leaders on other societal topics that were important within the school (e.g., climate change).

The experts organized 12 coaching sessions, in collaboration with the researchers. Overall, the HSCs reported having gained greater confidence in their changing roles as a result of these sessions, and they were particularly appreciative of the interaction with other HSCs. The coaching sessions allowed them to discuss difficulties that they had experienced in motivating leaders and facilitating participation, in addition to obtaining input on how to address difficulties, based on the experiences of others in similar situations. Detailed information on how the individual HSCs experienced their impact on creating leadership during the FLASH intervention is provided in Appendix III.

In addition to coaching sessions, HSCs mentioned that having more in-depth conversations with school leaders about their responsibilities in the process of creating a Healthy School and the need for their support to maintain the Healthy School had a positive impact on their leadership position. This provided them with more grounds to ask other colleagues to support the creation of a healthy school community. At the same time, the HSCs also experienced a lack of consistency in school leadership throughout the intervention, noting that changes in leadership are always accompanied by uncertainty regarding whether the new leader will be supportive of the Healthy School.

#### Strategy 2: Participatory school culture

- Adoption: the use of participatory methods.
- Implementation: the participation in Design Thinking and Photovoice.

Although all of the intervention schools used the participatory methods of Photovoice and Design Thinking, they adopted these methods differently (Table 3). Photovoice was applied to promote a participatory school culture among pupils. Based on experiences from the first year of the intervention that indicated that Photovoice could be used as a conversation starter in the curriculum as long as it was applied to a specific issue, the HSCs chose to apply it to engage pupils in designing an attractive physical environment that promotes healthy choices. In Schools 1, 2, and 4, this method was implemented in one or two second-year classes, each with approximately 25 *vmbo*-pupils during the second year of the intervention. Teachers and HSCs in these schools were particularly appreciative of the creativity and flexibility of Photovoice and the ideas that it generated for potential changes in the physical environment.

In collaboration with the local expert, the HSCs organized Design Thinking sessions. These sessions were attended by a number of colleagues, pupils and parents, but no support staff members (e.g., canteen employees) were willing to attend. Participant recruitment was most successful through using the informal network, presenting the work of pupils (e.g., Photovoice results), and providing incentives (e.g., offering a healthy meal). For example, in School 1, the HSC motivated people to join the Design Thinking session by serving healthy foods that pupils had prepared during a cooking workshop.

#### Strategy 3: Designing and implementing tailored health-promotion activities

- Adoption: creation of an action-plan based on Design Thinking session and initiation of new activities.
- Implementation: following through on action-plans and initiation of Healthy School activities.

During the Design Thinking sessions, stakeholders came up with multiple ideas for designing tailored health-promotion activities based on information gathered during interviews and Photovoice regarding the needs of the communities, and baseline information regarding the health behaviors of pupils. For implementation experiences per school regarding activities, see Appendix III. For example, stakeholders in School 2 prioritized the issue of many pupils going to a nearby supermarket to purchase unhealthy snacks. The school was open to improving their healthy canteen to make it a more attractive option for pupils. Stakeholders proposed a solution involving the introduction of a system of punch cards that parents could buy for their children, thereby providing pupils with easier and less expensive access to healthier options in the school canteen, as compared to the supermarket. After a session, all of the HSCs and one or two colleagues created concrete action plans based on the ideas proposed by stakeholders. The local experts and researchers particularly encouraged the HSCs and colleagues to use evidence-based intervention strategies and to include a communication plan to address visibility.

Schools carried out their action plans in the second half of the third intervention year. They were particularly successful in implementing activities that related to education or the physical environment. Examples included organizing a staircase-relay (School 4) and developing biology lessons about growing your own produce (School 1). The HSCs, teachers, and support staff members expressed that they had found it difficult to implement policy activities, as they had not felt that they had any mandate on overall school policies and the allocation of resources (e.g., money or time). In addition, stakeholders in Schools 1 and 2 mentioned that they had been unsure of how to increase visibility. In contrast, public-relations (PR) employees in Schools 3 and 4 had become involved by sharing news bulletins on their schools' communication platforms. For example, in School 3, the PR employee had prepared a news bulletin on social media when pupils received water bottles on World Water Day.

#### Strategy 4: Local networks

- Adoption: the availability of local experts and organization of coaching sessions.
- Implementation: initiated collaborations with local partners.

Throughout the FLASH intervention, it became evident that the HSCs assigned less priority to setting up local networks. Although they increasingly saw themselves as playing a mediating role between the local community and the school community, they did not feel ready to utilize these networks, and they were reluctant to approach possible partners, due to limited self-efficacy. All of the HSCs called upon PE colleagues to help them initiate collaborations with local sports clubs, as these teachers often had existing connections. Most of the other organizations that HSCs identified as potential partners were municipal institutions (e.g., youth services or spatial planning committees), but not local food retailers. Multiple stakeholders in Schools 1 and 4, both of which are located in small municipalities, reported successful partnerships with combination youth/health professionals (School 1) and care/sports coordinators (School 4). The HSCs in Schools 2 and 3, both of which are located in larger municipalities, were reluctant to initiate such collaborations, based on previous experiences in which such efforts had been time-consuming, difficult to maintain, and yielded little reward.

### Maintenance

- Maintenance was defined as the extent to which stakeholders intent to continue working with a community-based approach.

According to the experiences of stakeholders throughout the FLASH intervention, prior to the intervention, they had been more focused on organizing activities within each pillar of the Dutch Healthy School approach, paying less attention to setting up strong capacity-building processes. At the end of the intervention, stakeholders seemed to be more knowledgeable about why an integral approach and the involvement of more people offers greater potential than simply trying to meet the criteria of a particular Healthy School theme. Nonetheless, stakeholders in all schools agreed that the process of building community capacity takes time. In order to continue building capacity, the continuation of the HSCs role, as a linking pin and catalyst for the capacity-building process was considered essential. The HSCs and school leaders in all schools were searching for ways to continue this role without the additional FLASH hours after the end of the intervention. HSCs and other stakeholders involved in Healthy School activities expressed a need for continued support from experts in order to professionalize their leadership roles. For example, they reported needing support in finding ways to implement participatory methods into crowded curricula, ensuring proper representation of the community during participation, and identifying opportunities to start collaborations with food providers or municipal partners. Maintenance interviews with the local experts and managers of the municipal health service indicated that some aspects of the FLASH intervention (e.g., Photovoice, Design Thinking, coaching HSCs) have the potential to be embedded into existing tasks of supporting schools with the Dutch Healthy School approach. Proper training and time are important conditions to achieving this potential. Health promotion added that they also need to raise awareness of their own existing networks so that they can fully support in creating a Healthy School.

## Discussion

This study evaluated the impact of the FLASH intervention on community capacity, capacity-building processes, and the BMI and waist circumference of adolescents in four intervention schools. Community capacity improved across all intervention schools, but improvements varied between schools and between capacity-building strategies. Particular improvements were observed in terms of Strategy 1: leadership going from “vague awareness”/“pre-planning” to between “preparation” and “initiation”, Strategy 2: participatory school culture going from “vague awareness”/“pre-planning” to “preparation”, and Strategy 3A: the implementation of tailored activities supported by the community going from “preparation” to “initiation”. Limited increases were observed with regard to improving Strategy 3B: the visibility of activities (“vague awareness”/“pre-planning” to “pre-planning”), Strategy 3C: knowledge about local prevalence to prioritize activities, and Strategy 4: local networks (both from “pre-planning” to between “pre-planning and “preparation”). Building community capacity was experienced as a process that takes time. Having an appointed HSC was deemed essential for initiating processes of change and evoking participation. It was also noted, however, that HSCs needed to grow into their new leadership roles. All of the schools took important first steps in creating a healthy school community in which stakeholders have a sense of ownership. Results concerning BMI and waist circumference showed varying results with only small changes on BMI *z*-scores and inconsistent changes on waist circumference over time and between cohorts.

To our knowledge, only a few studies have used the CRC method to monitor change in community capacity to assess the impact of community-based interventions (15, 36, 37). Similar to FLASH, the interventions involved in these studies were aimed at strengthening collaborations and promoting ownership and resulted in similar increases in community capacity, with particular improvements in leadership. Comparable to these studies, the FLASH schools started the intervention with an overall capacity score between the vague awareness and pre-planning stages of readiness and increased to approximately the preparation stage over a three-year period. The finding that none of the schools increased to a capacity score above the initiation stage on any of the capacity-building strategies highlights the fact that the school communities might require more time or may need more support to structurally embed their efforts (15, 36, 37).

In line with other studies, we observed the most notable improvements in leadership (15, 36, 37). An essential element of the FLASH intervention was that HSCs were provided with time, which enabled them to take on a leadership role. This finding is in line with the advice of the Dutch Health School approach to spend (part of) a funding impulse that all Dutch schools can apply for on a similar task. Stakeholders in intervention schools responded well to the focus on building leadership as a first action for building a healthy school community. This finding is in line with previous research that concluded that knowledgeable, skilled and motivated leaders are key facilitators for sustainable implementation of public health interventions at school (8, 38). The results of this study indicate that both time and effort are required to build leadership within a community-based approach. The appointment of HSCs who adopt a capacity-building approach toward creating healthy school communities provides a linking pin between stakeholders and structures within the schools' dynamic context, in addition to serving as initiators of change. Given that the role may require a professional identity other than that of a teacher (39), HSCs need time to grow into this changing role. Experiences throughout the FLASH intervention suggest that it is especially important to be able to recognize opportunities within the wider context of a school (e.g., seeking collaborations on topics such as climate change), in addition to being able to act on these opportunities in a creative and flexible way. These experiences are in line with developments in the Schools for Health in Europe network, in which attention to the complexity of navigating the context is gaining momentum (40).

We also observed improvement in a participatory school culture. Our results suggest that enabling community members to share their ideas; keeping participation accessible, easy, and fun (e.g., through Design Thinking sessions); and giving a voice to community members contributed to a sense of ownership over the Healthy School. The experiences of stakeholders highlighted the reciprocal process of building this strategy. When participation is facilitated, activities are better received by community members, and this promotes positive awareness of the Healthy School and the willingness for further participation in the continuous developmental process of a Healthy School. Moreover, as demonstrated by our findings and emphasized in previous studies, it is imperative to have top-down support from members of the management, who set an example for the school culture to build this strategy (41, 42).

Concerning the implementation of health-promotion activities, we observed that schools mainly prioritize activities that they consider feasible and relatively easy or familiar to implement and that cause little discussion among community members. They do not necessarily consider whether activities are theoretically sound or evidence-based. To achieve behavioral change among pupils and improve health, it is important to assist schools in their efforts to obtain support for less popular, but evidence-based activities (43). At the same time, many studies have indicated that the evidence-based interventions that are available are not structurally adopted or followed with a high level of fidelity, such that they have only a limited impact on health behaviors (44–46). Although the Dutch Healthy School approach advocates the use of evidence-based interventions, it can be useful to let stakeholders prioritize fun but less evidence-based activities when schools are still in a stage of readiness where they are still working to improve awareness on healthy physical activity and dietary behavior.

We observed limited improvements in the local network strategy, as stakeholders were uncertain about their role to build networks. Previous studies have found mixed results on this strategy (15, 36, 37). However, these studies focused mainly on helping communities to apply for additional grants, unlike the FLASH intervention where we aimed at encouraging schools to build local and national partnerships that fit within the existing Dutch Healthy School approach. Given that the HSCs were still growing into their role of bringing people together within the school, they did not assign high priority to the complex process of creating networks around the school. Ideas for how local organizations in the fields of public health and education could offer support to schools varied between organizations.

The impact of the FLASH intervention on adolescent's BMI and waist circumference as secondary outcomes showed varying results and estimated differences between intervention and reference group were small. Particularly differences between both groups in waist circumference seemed inconsistent over time and between the different cohorts. We observed similar patterns in pupils behavior and attitudes in both groups. Because this intervention took place in real-life practice, schools in the reference group were allowed to implement health-promotion activities under the regular Dutch Healthy School approach. This may have had an effect on the difference found in anthropometric measures and lifestyle behaviors of pupils between groups. Additionally, this intervention specifically focused on the school community, whereas lifestyle behaviors of pupils are also influenced by activities outside the school setting, for example in the neighborhood or at sport clubs (47). Moreover, this study showed that the process of building community capacity among stakeholders takes time. Therefore it may have been too early to expect clear changes in behaviors and anthropometric measures of pupils within the study period. We did not collect information on pubertal stage and as this is strongly related to body adiposity, this could also have contributed to the inconsistent differences found in waist circumference between groups and between cohorts (48). Furthermore, the magnitude of measurement error in waist circumference has been reported to be varying (49).

### Strengths and limitations

One strength of this study is that it was performed in real-life practice. The process of building community capacity, as well as the evaluation of these processes, followed an adaptive approach to enable changes in the system to be captured and accommodated, and to allow for feedback, adjustments and emergent outcomes (22). We collected information on outcomes regarding the effectiveness of the FLASH intervention, on day-to-day processes, and on contextual factors that influence schools in practice. Because this approach allowed for the complexity of real-life situations, our study is consistent with the line of realistic evaluation (50, 51). The mixed-methods design (i.e., triangulation) and structured use of the RE-AIM elements helped to provide a true and complete picture of the impact of the FLASH intervention in complex real-life settings. Alongside the strengths of this study, it is important to acknowledge the subjective nature of assessing community capacity. We minimized this subjectivity by using two researchers to score interviews, each of whom kept a reflective diary in order to discuss potential subjective views. These reflections were also checked against the experiences that HSCs and local experts recorded in journals and meetings. In accordance with the CRC method we conducted between six and eight in-depth interviews per school (21, 29). Nevertheless, it could be that the participants responses still did not provide a complete picture of the community's readiness for change. We did include a diverse selection of key-stakeholders in different roles which helped obtain a multi-faceted picture of the process of capacity-building. Moreover, the rich data material contributed to a broad understanding of the specific community characteristics and dynamics. Although some efforts are being made to create an online validated questionnaire for the CRC method (52), these developments cannot yet be applied in the Dutch context. Given that our results demonstrate the promising impact of building community capacity, we recommend further research on how the principals of capacity building can be incorporated into the existing Dutch Healthy School approach, as well as on how a validated online tool could be developed for the Dutch context.

We evaluated the impact of the FLASH intervention at the school level (i.e., changes in community capacity) and at the individual level (i.e., pupils' BMI and waist circumference). It is important to note the limited number of schools and of pupils participating and the quasi-experimental study design. As is common in these types of studies (53, 54), we encountered challenges when involving *vmbo*-pupils in this effectiveness study (including obtaining parental consent). Additionally, we applied a quasi-experimental approach without randomization of schools, which might have resulted in residual confounding for the individual level outcomes.

### Implications for practice and research

This evaluation study contributed to a better understanding of the complexity of implementing health promotion in the whole school system, and demonstrate that contextual and dynamic processes, such as abrupt changes in pupil numbers or municipality plans for the physical environment, determine the implementation of the Healthy School approach. In order to support schools in the implementation of an integral whole-school approach, an important step for the sustainable implementation of the international Health Promoting School approach might be to concentrate on teaching stakeholders how to navigate these processes in and around their organizations. As indicated by our results, the focus on building community capacity and creating a broadly supported healthy school community helped schools to become more aware of their own contexts and dynamics, in contrast to a focus on whether an intervention (evidence-based or otherwise) is delivered as intended (55). This is also in contrast to the regular Dutch Healthy School approach where often only one stakeholder focusses on executing health-promotion activities. Stakeholders deemed the presence of a central coordinator charged with connecting people and opportunities within a school community essential to building community capacity, and they therefore suggested that this role should be structurally embedded in school policies. Given the finding that this role was new for the HSCs, future studies should focus on how to empower and support HSCs in this new role. In addition, further exploration of the role of local health-promotion professionals for supporting schools in their efforts to build community capacity would be worthwhile, particularly about the creation of local networks and the adaptation of evidence-based interventions to the local context. Stakeholders in this study looked at building local networks as an afterthought, while this could potentially also be a promising starting point to make schools feel more supported. It is therefore important to start a dialogue about who can take up which role around the school to best support schools in becoming a Health Promoting School.

## Conclusion

The results of this study highlight the potential of building community capacity to create healthy school communities which eventually might lead to a healthier body composition of pupils. Results also indicate that building community capacity is a highly dynamic and contextual process in which stakeholders must become acquainted with new leadership roles and responsibilities. In order to navigate this process, schools need support with improving communication, setting up local networks, and sustaining capacity-building efforts in school policy.

## Data availability statement

The raw data supporting the conclusions of this article will be made available by the authors upon reasonable request, without undue reservation.

## Ethics statement

The studies involving human participants were reviewed and approved by Medical Ethics Committee of Amsterdam UMC, VUmc location, reference number 2016.352. Written informed consent to participate in this study was provided by the participant and the participants' legal guardian/next of kin.

## Author contributions

BD, IV, CR, MR, and IS designed and executed the intervention and study. BD drafted the manuscript. IV, CR, MR, and IS provided input and feedback. MB advised on the experimental study and the analysis and provided feedback on the manuscript. All authors read and approved the final manuscript.

## Funding

This study was funded by a grant from major funding body the Netherlands Organization for Health Research and Development (ZonMw Grant No. 50-53105-98-033) and has undergone peer review by the funding body. The funding body did not play a role in the design, implementation, data collection, analysis, interpretation of data, or writing of the current manuscript and future manuscripts.

## Conflict of interest

The authors declare that the research was conducted in the absence of any commercial or financial relationships that could be construed as a potential conflict of interest.

## Publisher's note

All claims expressed in this article are solely those of the authors and do not necessarily represent those of their affiliated organizations, or those of the publisher, the editors and the reviewers. Any product that may be evaluated in this article, or claim that may be made by its manufacturer, is not guaranteed or endorsed by the publisher.

## Footnotes

^1^Explaining the Dutch education system: Pupils entering secondary education in the Netherlands are streamed according to aptitude into one of three forms of schooling: pre-vocational (*vmbo*), professional (*havo*), and pre-university (*vwo*). About 60% of all pupils are tracked into the *vmbo* stream, which consists of a four-year program (ages 12–16 years) intended as a route into secondary vocational education and training (*mbo*). After a two-year common basic curriculum, the *vmbo* track splits into sub-streams (i.e., “learning pathway” and “profile”), with selection according to ability, interests, and ambitions. Individual schools that provide more than one form of schooling (*vmbo/havo/vwo*), are classified as comprehensive schools.

## Abbreviations

FLASH intervention: Fit Lifestyle at School and at Home intervention
PA: Physical Activity
PE: Physical Education
HSC: Healthy School Coordinator
BMI: Body Mass Index
CRC method: Community Readiness to Change method.

## References

[1] Craigie AM Lake AA Kelly SA Adamson AJ Mathers JC. Tracking of obesity-related behaviours from childhood to adulthood: a systematic review. Maturitas. (2011) 70:266–84. 10.1016/j.maturitas.2011.08.005 21920682

[2] Singh AS Mulder C Twisk JW Van Mechelen W Chinapaw MJ. Tracking of childhood overweight into adulthood: a systematic review of the literature. Obesity reviews. (2008) 9:474–88. 10.1111/j.1467-789X.2008.00475.x 18331423

[3] Katz D. O'connell M, Njike VY, Yeh M, Nawaz H. Strategies for the prevention and control of obesity in the school setting: systematic review and meta-analysis. Int J Obesity. (2008) 32:1780–9. 10.1038/ijo.2008.158 19079319

[4] Young I St Leger L Buijs G. School health promotion: evidence for effective action. Background Paper SHE Factsheet. (2013) 2:1–23.

[5] Turunen H Sormunen M Jourdan D von Seelen J Buijs G. Health promoting schools—a complex approach and a major means to health improvement. Health Promot Int. (2017) 32:177–84. 10.1093/heapro/dax001 28384373

[6] Doak C Visscher T Renders C Seidell J. The prevention of overweight and obesity in children and adolescents: a review of interventions and programmes. Obesity Rev. (2006) 7:111–36. 10.1111/j.1467-789X.2006.00234.x 16436107

[7] Scheirer MA. Is Sustainability possible? A review and commentary on empirical studies of program sustainability. Am J Eval. (2005) 26:320–47. 10.1177/1098214005278752

[8] Rowling L Samdal O. Filling the Black Box of Implementation for Health-Promoting Schools. Health Educ. (2011) 111:347–62. 10.1108/09654281111161202

[9] King L Gill T Allender S Swinburn B. Best practice principles for community-based obesity prevention: development, content and application. Obes Rev. (2011) 12:329–38. 10.1111/j.1467-789X.2010.00798.x 20880111

[10] Swinburn B Malakellis M Moodie M Waters E Gibbs L Millar L. Large Reductions in child overweight and obesity in intervention and comparison communities 3 years after a community project. Pediatr Obes. (2014) 9:455–62. 10.1111/j.2047-6310.2013.00201.x 24203373

[11] Rosas SR. Systems thinking and complexity: considerations for health promoting schools. Health Promot Int. (2017) 32:301–11. 10.1093/heapro/dav109 26620709

[12] Hawe P Noort M King L Jordens C. Multiplying health gains: the critical role of capacity-building within health promotion programs. Health Policy. (1997) 39:29–42. 10.1016/S0168-8510(96)00847-0 10164903

[13] Bergeron K Abdi S DeCorby K Mensah G Rempel B Manson H. Theories, models and frameworks used in capacity building interventions relevant to public health: a systematic review. BMC Public Health. (2017) 17:914. 10.1186/s12889-017-4919-y 29183296 PMC5706342

[14] Hoyle TB Samek BB Valois RF. Building capacity for the continuous improvement of health-promoting schools. J School Health. (2008) 78:1–8. 10.1111/j.1746-1561.2007.00259.x 18177294

[15] Millar L Robertson N Allender S Nichols M Bennett C Swinburn B. Increasing community capacity and decreasing prevalence of overweight and obesity in a community based intervention among australian adolescents. Prev Med. (2013) 56:379–84. 10.1016/j.ypmed.2013.02.020 23485797

[16] Liberato SC Brimblecombe J Ritchie J Ferguson M Coveney J. Measuring capacity building in communities: a review of the literature. BMC Public Health. (2011) 11:850. 10.1186/1471-2458-11-850 22067213 PMC3229539

[17] Leurs MTW Schaalma HP Jansen MWJ Mur-Veeman IM Leger LH de de Vries N Development of a collaborative model to improve school health promotion in the netherlands. Health Promot Int. (2005) 20:296–305. 10.1093/heapro/dai004 15797902

[18] RIVM. Zoektool Gezonde Scholen - Overzicht Vo Scholen Met Vignet Gezonde School Per Themacertificaat. (2019). Available online at: https://mijngezondeschool.nl/zoektool (cited January 10, 2020).

[19] Boot NM de Jongh DM Leurs MT de Vries NK. Gezonde School Als Methode Voor Ggd'en Bij De Invoering Van Schoolgezondheidsbeleid. Tijdschrift voor gezondheidswetenschappen. (2011) 89:222–8. 10.1007/s12508-011-0075-4

[20] van Dongen BM Ridder MAM Steenhuis IHM Renders CM. Background and evaluation design of a community-based health-promoting school intervention: fit lifestyle at school and at home (flash). BMC Public Health. (2019) 19:784. 10.1186/s12889-019-7088-3 31221106 PMC6585041

[21] Plested BA Edwards RW Jumper-Thurman P. Community Readiness: A Handbook for Successful Change. Research T-ECfP, editor Fort Collins: Tri-Ethnic Center for Prevention Research (2006).

[22] Eoyang G Oakden J. Adaptive evaluation. Emerg Complex Organ. (2016) 18:1–14. 10.emerg/10.17357.e5389f5715a734817dfbeaf25ab335e5

[23] Glasgow RE Vogt TM Boles SM. Evaluating the public health impact of health promotion interventions: the re-aim framework. Am J Public Health. (1999) 89:1322–7. 10.2105/AJPH.89.9.1322 10474547 PMC1508772

[24] Glasgow RE Harden SM Gaglio B Rabin B Smith ML Porter GC. Re-aim planning and evaluation framework: adapting to new science and practice with a 20-year review. Front Public Health. (2019) 7:64. 10.3389/fpubh.2019.00064 30984733 PMC6450067

[25] RIVM. De Gezonde School—Over. Ons: RIVM. Available online at: https://www.gezondeschool.nl/over-ons (cited January 22, 2019).

[26] Cutler DM Lleras-Muney A editors. Education and Health: Evaluating Theories and Evidence. (2006). Cambridge: National bureau of economic research: National bureau of economic research.

[27] Ministry of Education Culture Science. Pre-Vocational Seconary Education (Vmbo) The Hague: Government of the Netherlands. (2020). Available online at: https://www.government.nl/topics/secondary-education/pre-vocational-secondary-education-vmbo (cited August 21, 2020).

[28] Unit DE. The Education System in the Netherlands. (2007). The Hague: Ministry of Education, Culture and Science.

[29] Edwards RW Jumper-Thurman P Plested BA Oetting ER Swanson L. Community readiness: research to practice. J Commun Psychol. (2000) 28:291–307. 10.1002/(SICI)1520-6629(200005)28:3&lt

[30] Kehl M Brew-Sam N Strobl H Tittlbach S Loss J. Evaluation of community readiness for change prior to a participatory physical activity intervention in Germany. Health Promot Int. (2021) 36:ii40–52. 10.1093/heapro/daab161 34905609 PMC8670622

[31] Evenhuis I Wezenbeek N Vyth E Veldhuis L Poelman M Wolvers D. Development of the ‘canteen scan': an online tool to monitor implementation of healthy canteen guidelines. BMC Public Health. (2018) 18:1109. 10.1186/s12889-018-5974-8 30200919 PMC6131796

[32] Fleuren M Paulussen T Van Dommelen P Van Buuren S. Measurement Instrument for Determinants of Innovations (Midi). (2014). Leiden: TNO. 10.1093/intqhc/mzu060 PMC4195468 24951511

[33] Fredriks AM Van Buuren S Burgmeijer RJ Meulmeester JF Beuker RJ Brugman E. Continuing positive secular growth change in the Netherlands 1955–1997. Pediatr Res. (2000) 47:316–23. 10.1203/00006450-200003000-00006 10709729

[34] Janssen EH Singh AS van Nassau F Brug J van Mechelen W Chinapaw MJ. Test–retest reliability and construct validity of the doit (Dutch Obesity Intervention in Teenagers) questionnaire: measuring energy balance-related behaviours in dutch adolescents. Public Health Nutr. (2014) 17:277–86. 10.1017/S1368980012005253 23217249 PMC10282246

[35] CBS. Wat Is Het Verschil Tussen Een Westerse En Niet-Westerse Persoon Met Een Migratie Achtergrond?: CBS. (2021). Available online at: https://www.cbs.nl/nl-nl/faq/specifiek/wat-is-het-verschil-tussen-een-westerse-en-niet-westerse-allochtoon-#:~:text=Niet%2Dwesters%3A,het%20dossier%20Migratie%20en%20Integratie (cited February 12, 2021).

[36] Whelan J Love P Millar L Allender S Morley C Bell C. Rural community moves closer to sustainable obesity prevention-an exploration of community readiness pre and post a community-based participatory intervention. BMC Public Health. (2019) 19:1–9. 10.1186/s12889-019-7644-x 31666042 PMC6820900

[37] Heath E Sanon V Mast DK Kibbe D Lyn R. Increasing community readiness for childhood obesity prevention: a case study of four communities in Georgia. Health Promot Pract. (2020) 22:676–684. 10.1177/1524839920917127 32406260

[38] Herlitz L MacIntyre H Osborn T Bonell C. The sustainability of public health interventions in schools: a systematic review. Implement Sci. (2020) 15:1–28. 10.1186/s13012-019-0961-8 31906983 PMC6945701

[39] Jourdan D Simar C Deasy C Carvalho GS McNamara PM. School health promotion and teacher professional identity. Health Educ. (2016) 116:106–122. 10.1108/HE-07-2014-0078

[40] Bartelink N Bessems K. Health Promoting Schools in Europe—State of the Art. Denmark (2019).

[41] Dadaczynski K Paulus P. Healthy principals–healthy schools? A neglected perspective to school health promotion. Schools for Health and Sustainability. (2015). Berlin: Springer. p. 253–73.

[42] Larsen T Samdal O. Facilitating the implementation and sustainability of second step. Scand J Educ Res. (2008) 52:187–204. 10.1080/0031383080191582035441007

[43] Council NB. Public Health: Ethical Issues. London: Nuffield Council on Bioethics. (2007).

[44] van Nassau F Singh AS Cerin E Salmon J van Mechelen W Brug J. The Dutch obesity intervention in teenagers (doit) cluster controlled implementation trial: intervention effects and mediators and moderators of adiposity and energy balance-related behaviours. Int J Behav Nutr Phys Activity. (2014) 11:1–11. 10.1186/s12966-014-0158-0 25539582 PMC4304621

[45] Martens M van Assema P Paulussen T Schaalma H Brug J. Krachtvoer: Process evaluation of a Dutch programme for lower vocational schools to promote healthful diet. Health Educ Res. (2006) 21:695–704. 10.1093/her/cyl082 16945986

[46] Martens MK Van Assema P Paulussen TG Van Breukelen G Brug J. Krachtvoer†: effect evaluation of a dutch healthful diet promotion curriculum for lower vocational schools. Public Health Nutr. (2008) 11:271–8. 10.1017/S1368980007000298 17605839

[47] Bartelink N. Evaluating Health Promotion in Complex Adaptive School Systems: The Healthy Primary School of the Future (2019) 9–22.

[48] Adami F Benedet J Takahashi LAR da Silva Lopes A da Silva Paiva L de Vasconcelos FdAG. Association between Pubertal Development Stages and Body Adiposity in Children and Adolescents. Health Qual Life Outcomes. (2020) 18:1–9. 10.1186/s12955-020-01342-y 32252769 PMC7137486

[49] Verweij LM Terwee CB Proper KI Hulshof CT van Mechelen W. Measurement error of waist circumference: gaps in knowledge. Public Health Nutr. (2013) 16:281–8. 10.1017/S1368980012002741 22626254 PMC10271771

[50] Blamey A Mackenzie M. Theories of change and realistic evaluation: peas in a pod or apples and oranges? Evaluation. (2007) 13:439–55. 10.1177/135638900708212933389043

[51] Robson A. The potential contribution of realistic evaluation to small-scale community interventions. Commun Pract. (2008) 81:25. 18834025

[52] Kostadinov I Daniel M Stanley L Cargo M. Assessing community readiness online: a concurrent validation study. BMC Public Health. (2015) 15:1–6. 10.1186/s12889-015-1953-5 26135737 PMC4489112

[53] Tigges BB. Parental consent and adolescent risk behavior research. J Nurs Scholarship. (2003) 35:283–9. 10.1111/j.1547-5069.2003.00283.x 14562498

[54] Fargas-Malet M McSherry D Larkin E Robinson C. Research with children: methodological issues and innovative techniques. J Early Childhood Res. (2010) 8:175–92. 10.1177/1476718X09345412

[55] van Nassau F Singh AS van Mechelen W Brug J Chinapaw MJ. Implementation evaluation of school-based obesity prevention programmes in youth; how, what and why? Public Health Nutr. (2015) 18:1531–4. 10.1017/S1368980014002778 25491188 PMC10271618

