# Supplementary material for: Building community capacity to stimulate physical activity and dietary behavior in Dutch secondary schools: Evaluation of the FLASH intervention using the REAIM framework
Source: Front Public Health. 2022 Aug 3;10:926465. doi: 10.3389/fpubh.2022.926465 (PMC9381984; doi:10.3389/fpubh.2022.926465)
Supplement: Supplementary file 1 [file Data_Sheet_1.docx]

**Appendix I – Operationalization and descriptive statistics for behavioral outcomes**

|  |  |  | **Intervention** | | | | **Reference** | | | |
| --- | --- | --- | --- | --- | --- | --- | --- | --- | --- | --- |
| **Outcome** | **Items** | **Operationalization** | **T1** | **T2** | **T3** | **T4** | **T1** | **T2** | **T3** | **T4** |
|  |  |  | N=81 | N=138 | N=201 | N=127 | N=93 | N=204 | N=303 | N=218 |
| **Dutch Norm Healthy PA** | - How many days do you exercise for at least one hour? | Days a week (0–7) | 4.8  (1.9) | 5.3  (1.9) | 5.4  (1.9) | 5.7  (1.9) | 5.1 (1.9) | 5.3 (2.1) | 5.3 (2.1) | 5.7 (1.9) |
| **Screen time behavior** | - How many days do you play games (1 item)/use social media (1 item)/watch programs on TV or other screens (1 item)? - How much time do you spend playing games (1 item)/using social media (1 item)/watching programs on TV or other screens (1 item) on an average weekday? | Number of days (0.5–6.5) x number of hours (0–6) added for games/social media/programs, divided by 7 to calculate average screen time in hours a day | 3.7 (2.1) | 3.5 (2.2) | 4.1 (2.3) | 4.3 (2.4) | 3.8 (2.2) | 4.1 (2.4) | 4.2 (2.4) | 4.5 (2.3) |
| **Water consumption** | - How many days a week do you drink water? - How many glasses of water do you drink on an average day? | Number of days (0–7) x average number of glasses a day (0–7), divided by 7 to calculate average water consumption in glasses a day | 2.3 (1.8) | 2.6 (2.0) | 2.6 (1.8) | 3.0  (2.1) | 2.7 (1.8) | 2.8 (1.9) | 3.0 (1.9) | 2.8 (1.8) |
| **SSB consumption** | - How many days a week do you drink soda (1 item)/fruit juice (1 item)? - On an average school day, how much soda/fruit juice do you drink from a) small packages (200ml) and b) large packages (440ml)? | Adding soda and fruit juice consumption in ml, calculated for each by adding ml of small and large packages (0–7) x by number of days (0–7), divided by 7 to calculate average soda/juice consumption in ml a day | 522 (647) | 520 (771) | 539 (641) | 516 (625) | 594 (721) | 534 (567) | 567 (626) | 539 (667) |
| **Breakfast consumption** | - How many days a week do you eat breakfast? | Days a week (0–7) | 6.3 (1.8) | 6.0 (2.0) | 6.0 (2.0) | 6.0 (2.0) | 6.4 (1.4) | 6.1 (2.0) | 5.8 (2.2) | 5.7 (2.3) |
| **Fruit consumption** | - How many days a week do you eat fruit? | Days a week (0–7) | 4.2 (2.3) | 4.3 (2.3) | 4.3 (2.3) | 4.2 (2.3) | 4.1 (2.3) | 4.4 (2.3) | 4.1 (2.3) | 4.0 (2.4) |
| **Vegetable consumption** | - How many days a week do you eat vegetables? | Days a week (0–7) | 5.6 (1.3) | 5.6 (1.4) | 5.3 (1.5) | 5.2 (1.6) | 5.6 (1.4) | 5.5 (1.7) | 5.4 (1.6) | 5.4 (1.6) |
| **Snack consumption** | - How many days a week do you eat snacks (1 item)/candy (1 item)? - On an average school day, how many snacks (1 item)/how much candy (1 item) do you eat? | Adding snack and candy consumption, calculated for each by adding number of days (0–7) x servings a day (0–5) divided by 7 to calculate average snack consumption in servings a day | 1.0 (2.0) | 1.1 (1.3) | 1.3 (1.4) | 1.3 (1.4) | 1.2 (1.3) | 1.3 (1.4) | 1.3 (1.2) | 1.2 (0.9) |
| **Attitude** | - What do you think of drinking sugar-sweetened beverages? | Scoring each topic on 5-point scale answering the statement: I find x ..   1. Very good 2. Good 3. Not good/not bad 4. Bad 5. Very bad | 2.70 (.732) | 2.63 (.705) | 2.61 (.738) | 2.71 (.775) | 2.61 (.847) | 2.66 (.763) | 2.64 (.710) | 2.69 (.775) |
|  | - What do you think of drinking energy drinks? |  | 3.84 (1.042) | 3.65 (1.019) | 3.83 (1.116) | 3.63 (1.129) | 3.70 (1.159) | 3.73 (1.065) | 2.82 (.990) | 3.74 (.935) |
|  | - What do you think of drinking fruit juice? |  | 2.02 (0.689) | 2.22 (.832) | 2.25 (.765) | 2.33 (.852) | 2.04 (.908) | 2.16 (.765) | 2.15 (.754) | 2.32 (.856) |
|  | - What do you think of consuming snacks? |  | 2.78 (.791) | 2.74 (.745) | 2.73 (.751) | 2.88 (.732) | 3.00 (.780) | 2.82 (.754) | 2.90 (.798) | 2.83 (.738) |
|  | - What do you think of consuming candy? |  | 2.80 (.714) | 2.79 (.766) | 2.84 (.839) | 3.05 (.859) | 3.06 (.734) | 2.96 (.755) | 2.99 (.754) | 2.92 (.750) |
|  | - What do you think of drinking water? |  | 1.53 (.726) | 1.48 (.726) | 1.48 (.752) | 1.42 (.659) | 1.41 (.811) | 1.38 (.629) | 1.42 (.667) | 1.39 (.704) |
|  | - What do you think of eating breakfast every day? |  | 1.35 (.655) | 1.43 (.762) | 1.30 (.640) | 1.48 (.742) | 1.27 (.678) | 1.31 (.611) | 1.36 (.671) | 1.42 (.668) |
|  | - What do you think of consuming fruit? |  | 1.53 (.743) | 1.50 (.652) | 1.54 (.683) | 1.52 (.676) | 1.40 (.645) | 1.42 (.651) | 1.49 (.646) | 1.48 (.693) |
|  | - What do you think of being physically active for one hour a day? |  | 1.73 (.908) | 1.71 (.864) | 1.67 (.753) | 1.63 (.812) | 1.57 (.682) | 1.48 (.734) | 1.64 (.834) | 1.67 (.841) |
|  | - What do you think of gaming every day? |  | 3.28 (.912) | 3.11 (1.012) | 2.97 (1.014) | 3.01 (1.039) | 3.35 (1.158) | 3.18 (1.119) | 3.05 (1.004) | 3.04 (.971) |
|  | - What do you think of using social media every day? |  | 2.35 (.793) | 2.38 (.784) | 2.30 (.778) | 2.27 (.707) | 2.42 (.901) | 2.43 (.796) | 2.42 (.798) | 2.29 (.727) |
|  | - What do you think of watching shows/videos every day on TV or computer/tablet/phone? |  | 2.62 (.768) | 2.58 (.752) | 2.46 (.771) | 2.40 (.737) | 2.62 (.806) | 2.54 (.720) | 2.56 (.783) | 2.47 (.706) |
|  | - What do you think of going to school by bike or by foot every day? |  | 1.44 (.707) | 1.59 (.668) | 1.54 (.683) | 1.60 (.881) | 1.43 (.579) | 1.54 (.753) | 1.58 (.759) | 1.60 (.779) |
