# Supplementary material for: Building community capacity to stimulate physical activity and dietary behavior in Dutch secondary schools: Evaluation of the FLASH intervention using the REAIM framework
Source: Front Public Health. 2022 Aug 3;10:926465. doi: 10.3389/fpubh.2022.926465 (PMC9381984; doi:10.3389/fpubh.2022.926465)
Supplement: Supplementary file 2 [file Data_Sheet_2.docx]

**Appendix II – Contextual factors and characteristics of intervention schools^[[1]](#footnote-1)^ throughout the FLASH intervention**

**School 1**

- Comprehensive school, located in a rural area. All *vmbo* sub-streams are offered, but pupils choosing the “profile” sub-stream must change schools after two years. The “learning pathway” sub-streams are offered to completion (4 years).
- Pupil enrollment in all levels declined throughout the intervention.
- As this school is a comprehensive school, the intervention was aimed predominantly at *vmbo* pupils, but pupils from other levels were not excluded from actions/activities that changed facilities or systems within the school, as they are shared by all pupils.
- A nutrition vignette^[[2]](#footnote-2)^ was obtained at the start of the intervention, but school leaders also noted that it was not possible to continue this certification, as they were unable to comply with the Healthy School canteen regulations, due to their small capacity. The physical activity vignette had not been obtained at the start of the intervention, and the school had no intention of obtaining it. Although the school was already in compliance with most criteria, they did not feel that they could meet the criterion of structural cooperation with external sport organizations, due to the school’s rural location.

**School 2**

- Comprehensive school, located in a city center. Only the “learning pathway” sub-streams are offered.
- Total pupil enrollment increased throughout the intervention, but *vmbo* enrollment remained consistent.
- As this school is a comprehensive school, the intervention was aimed predominantly at *vmbo* pupils, but pupils from other levels were not excluded from actions/activities that changed facilities or systems within the school, as they are shared by all pupils. Due to the increase in the pupil population, a temporary additional location was opened in the third year of the intervention, with *vmbo* pupils being concentrated at this location. As this location was temporary, no canteen, school yard, or similar facilities were present.
- The school is classified as a “sports school,” as it offers more extensive PE than other schools. As a result, the school had already obtained the physical activity vignette at the beginning of the intervention. Although the school maintained this vignette throughout the intervention, difficulties were expressed with regard to finding appropriate sites for exercising, due to its location in a city center. The school was also already in the process of obtaining the nutrition vignette. Although it already had a Healthy School canteen and water tap, it did not have a structural school policy on nutrition. At the end of the intervention, the school had started working on the policy, but had not completed it.

**School 3**

- Exclusively offers the “profiles” sub-streams of the *vmbo* track. Located in a city center.
- Pupil enrollment remained consistent throughout the intervention.
- The school experienced a merging of the PrO and *vmbo* streams within its building. The school leaders decided to aim the intervention only at *vmbo* pupils as education and facilities were predominantly separated in different parts of the building (such as separate school-yards and -canteens). Due to this merging, the school building underwent renovations (scheduled before the start of FLASH) that changed the physical environment (mainly hallways and schoolyard)
- At the start of the intervention, the school had obtained the physical activity vignette, but not the nutrition vignette. Although the physical activity vignette was maintained throughout the intervention, difficulties were noted with regard to finding appropriate sites for exercising, due to the school’s location in a city center. The school was also already working on the nutrition vignette, but the lack of a water tap for pupils prevented them from obtaining it. Throughout the intervention, the school also made changes to the Healthy School canteen even though it met the criteria, in order to generate greater support among support staff and pupils.

**School 4**

- Comprehensive school, located in a rural area. At the start of the intervention, a new school location with separate facilities was opened, which exclusively offers the “profiles” sub-streams of the *vmbo* track. The intervention was specifically aimed at this location, as it operated as a separate school community.
- Pupil enrollment remained consistent throughout the intervention.
- The new location shared some of its facilities (e.g., canteen, school yard) with another secondary pre-vocational school and a vocational education and training (VET) school. The intervention was nevertheless aimed predominantly at pupils, parents, and teachers of the participating school, as each of the schools made its own decisions with regard to their Healthy School approach.
- As this location was new, no vignettes had been obtained at the start of the intervention. The school nevertheless met all of the criteria for the physical activity vignette, which was subsequently obtained during the course of the intervention. Although the school aimed to obtain the nutrition vignette as well during the intervention, it encountered difficulties in realizing the criteria, particularly with regard to a Healthy School canteen and water taps. This was mainly due to the fact that the school did not have full control over the design of these physical facilities and needed to coordinate with the other two schools. At the end of the intervention, the school canteen had received Healthy School certification, and plans had been outlined to install water taps for the following school year.

1. **Explaining the Dutch education system:** Pupils entering secondary education in the Netherlands are streamed according to aptitude into one of four forms of schooling: practical (PrO), pre-vocational (*vmbo*), professional (*havo*), and pre-university (*vwo*). About 60% of all pupils are tracked into the *vmbo* stream, which consists of a four-year program (ages 12–16 years) intended as a route into upper secondary vocational education and training (*mbo*). After a two-year common basic curriculum, the *vmbo* track splits into sub-streams (i.e., “learning pathway” and “profile”), with selection according to ability, interests, and ambitions. Individual schools that provide more than one form of schooling (*vmbo/havo/vwo*), are classified as comprehensive schools. [↑](#footnote-ref-1)
2. The **Dutch Healthy School approach:** This is the translation of the integral whole-school approach, as advocated by the WHO. The approach is built on a system of theme vignettes and four integral pillars (health education, health policy, a healthy social and physical environment, and having a system of signaling and referral). Schools can earn vignettes on several themes (e.g., physical activity or nutrition) if they can demonstrate that they pay structural attention to encouraging healthy behavior, and if they have evidence-based (or other) activities for each pillar. [↑](#footnote-ref-2)
