# Supplementary material for: Building community capacity to stimulate physical activity and dietary behavior in Dutch secondary schools: Evaluation of the FLASH intervention using the REAIM framework
Source: Front Public Health. 2022 Aug 3;10:926465. doi: 10.3389/fpubh.2022.926465 (PMC9381984; doi:10.3389/fpubh.2022.926465)
Supplement: Supplementary file 3 [file Data_Sheet_3.docx]

**Appendix III – Experiences with implementation at each school**

**School 1:** The HSC reported having become more comfortable over time with the role of identifying leaders. After the first year of the intervention, a new manager was appointed in the school. This was beneficial for the HSC, as the new manager was intrinsically motivated to promote health among pupils. As this manager made it clear in regular contact moments (e.g., team meetings, parent evenings) that health was an important topic in the school, the HSC felt more justified in asking others to take on leadership roles. A PE teacher was already fulfilling a leadership role in promoting physical activity and continued to do so. A new biology teacher was intrinsically motivated to promote healthy behavior when she started at the school, and she became more involved in setting up new activities. The HSC also tried to put the canteen employee in a leadership role, but later realized that this person lacked capabilities for this role. Instead, a vocal and well-connected janitor become a spokesperson between various parties (e.g., pupils, teachers, managers) for creating an attractive food environment. Both the pupil- and parent councils have designated health as a regular topic on their agendas, as encouraged by the school manager and janitor.

In the Design Thinking session, community members decided to focus on reducing sedentary behavior during school hours. The most promising activity was deemed to be acquiring standing desks and activity chairs that pupils could use during biology class, accompanied by education about sedentary behavior and a policy concerning when specific materials are to be introduced. Although the implementation of this action plan was delayed, the biology teacher implemented a different idea from the Design Thinking session: a new curriculum focusing on having pupils grow healthy foods in the greenhouse. It was possible to implement both plans, as the second idea did not require any additional resources.

**School 2:** The HSC reported having learned many additional skills throughout the course of the intervention. As reported by this HSC, the school director and team manager had already been supportive at the start of the intervention, but together they had learned to achieve a better balance between their responsibilities. For example, school leaders have greater responsibility for creating policy, while the role of the HSC focuses more on coordinating activities and motiving other stakeholders. The HSC also indicated that he had learned the importance of having someone to bounce ideas off and to hold him accountable for continuing to work on the assigned tasks. To this end, he worked with a close colleague (both were PE teachers). The HSC initiated a partnership with a biology teacher who was leading a working group on climate change and had taken on responsibility for reforming the pupil council. This made it possible to connect the topic of health to other societal themes and place it on the agenda of the pupil council. The HSC also enhanced his partnership with the cafeteria employee as a leader of the food environment. This employee became more aware of their potential role and therefore became more involved in canteen-related activities. Aside from the reform of the pupil-council, the HSC found it difficult to identify additional leaders among pupils and parents in the *vmbo* stream, as these stakeholders moved to a different building in Year 3.

In the Design Thinking session, community members decided to focus on reducing purchases of unhealthy snacks in a nearby supermarket. The most promising activity was deemed to involve setting up a punch-card card system to make it more attractive, easier, and less expensive to purchase products in the Healthy School canteen, in addition to adopting a policy and rules about food in the school. This action plan was carried out, but it did not have the intended impact, as little attention was paid to promoting the punch-card system, and *vmbo*-pupils (the intended target group) were transferred to a different location that did not have a canteen.

**School 3:** The first HSC (Years 1–2) reported having felt increasingly motivated, but also acknowledged a number of organizational constraints. Given that this coordinator also fulfilled a managerial role, he did not feel that he had the full support of other managers with regard to the HSC role, while also perceiving a certain distance between himself and teaching colleagues. The situation was exacerbated after the first year of the intervention, when a new school director was appointed, who had little motivation for the topic of health. The HSC also reported not always having been able to spend the allotted FLASH hours due to other responsibilities. He nevertheless succeeded in setting up a working group with two other teachers (biology and economics). As this school has a system that requires every teacher to become involved with an extracurricular topic, facilitated by task hours, these teachers also received some time for this role. The HSC also tried to involve the canteen employee, who ultimately could not be motivated. The HSC then started to involve a PR employee who was intrinsically motivated with regard to the topic of health. The second HSC (Year 3) continued this involvement, expanding this employee’s role in organizing specific activities. The second HSC did not have a managerial role and reported having felt more supported by the management team. In particular, one manager had taken on the responsibility of facilitating the HSC and become a person the HSC could bounce ideas off. Although it was more difficult to assign a leadership role to parents and pupils the HSC did try to actively involve pupils who were in his own class.

The HSC decided to focus on promoting water consumption by providing pupils with water bottles, accompanied by making the water tap stand out with nudges and providing education about sugary drinks. Water bottles were distributed during an event that received PR exposure throughout the community. No additional activities were carried, due the unavailability of an HSC.

**School 4:** The first HSC (Year 1) encountered difficulties in identifying leadership, as everyone was preoccupied with becoming accustomed to a new school structure. She did not feel supported by school leaders, as they were also still finding their place. This influenced her motivation and sense of capability. The second HSC (Years 1–2) seemed more assertive under similar circumstances. Despite continuous leadership changes and organizational tensions in the school, this HSC engaged in conversations with school managers. He noted that this was easier with managers who were intrinsically motivated for health than it was with those who were less motivated. The HSC was able to involve a few teachers as leaders of the Healthy School community (a biology teacher and two health and well-being teachers). In addition, a PR employee became a leader for increasing familiarity among colleagues and parents. The HSC experienced more difficulty with identifying leadership among pupils and parents, as no official parent or pupil council had yet been established for the new location.

In the Design Thinking session, community members decided to focus on promoting physical activity during school hours. The most promising activity was deemed to involve organizing a recurring staircase challenge, which uses the most prominent feature of the building, accompanied by the installation of a water tap and an educational module in which pupils learn about heart rate and the implications of physical activity for their own lives. This action plan was carried out, albeit with minor adjustments. School leaders blocked access to most prominent staircase, but the HSC shifted the challenge to outside steps and staircases on which pupils are usually not allowed. An added bonus came from a redistribution of school budget, which meant that the water tap could be funded from other resources. This left extra implementation budget for the educational module.
